# Nanomedicine breakthroughs overcoming pancreatic cancer drug resistance through precision nano-interventions

**DOI:** 10.1039/d5na00513b

**Published:** 2025-07-29

**Authors:** Linjia Peng, Yanfeng Liang, Xiaonan Guo, Qiuli Zhang, Zixuan Gao, Xinxin Kong, Haiting Zhang, Binyu Zhu, Daxiang Cui

**Affiliations:** a The First Afffliated Hospital of Henan University No. 357, Ximen Street Kaifeng 475004 China dxcui@sjtu.edu.cn

## Abstract

Pancreatic ductal adenocarcinoma (PDAC) is one of the most lethal malignancies, primarily due to its rapid acquisition of drug resistance and the complex tumor microenvironment. Conventional cancer therapies, including chemotherapy and radiotherapy, often fail to elicit durable responses because PDAC cells exhibit both intrinsic and extrinsic resistance, in which the intrinsic resistance is driven by genetic mutations, epigenetic alterations, overexpression of efflux transporters, and the presence of cancer stem cells while the extrinsic resistance is mediated by a dense desmoplastic stroma, hypovascularity, and immunosuppressive cellular components. This review comprehensively analyzes these multifactorial resistance mechanisms and examines cutting-edge nanotechnology-based strategies designed to circumvent them. We discuss the design of intelligent, stimuli-responsive nanocarriers, including pH-sensitive, redox-sensitive, and enzyme-activated systems that enable spatiotemporally controlled drug release, thereby enhancing drug accumulation within tumor cells while minimizing systemic toxicity. Additionally, advances in surface functionalization and active targeting strategies, such as the use of ligand-conjugated nanoparticles, are highlighted for their role in enhancing selective delivery to both the bulk tumor cells and therapy-resistant cancer stem cell populations. Mechanistic insights are provided into how these nanomedicine interventions bypass traditional resistance pathways by facilitating intracellular drug delivery, co-delivering combination therapies, and modulating the tumor microenvironment to enhance therapeutic efficacy. These innovative strategies offer promising avenues to overcome drug resistance in PDAC, potentially transforming therapeutic outcomes for this aggressive disease.

## Abstract 2

Stimuli-responsive nanocarriers tailored to bypass efflux pumps, target cancer stem cells, and remodel the stroma overcome PDAC resistance and enhance therapeutic efficacy.

## Graphical abstract

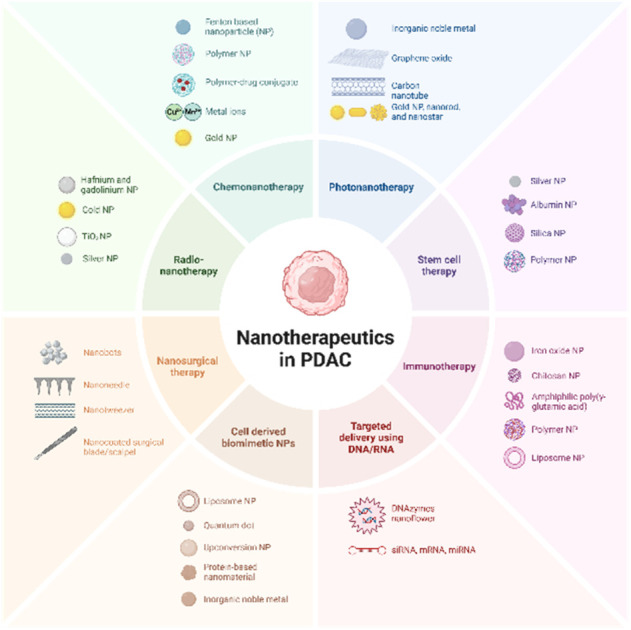

## 1. Introduction

Pancreatic ductal adenocarcinoma (PDAC) is one of the most lethal human malignancies, with a five-year survival rate below 10%.^1,2^ This dismal prognosis reflects its typically late-stage diagnosis, aggressive metastatic spread, and a tumor microenvironment that undermines conventional therapies.^3^ Unlike malignancies amenable to early detection by targeted agents, PDAC is often identified when surgical resection is no longer possible,^4,5^ leaving systemic chemotherapy and radiotherapy as the mainstays—modalities that frequently fail to produce durable responses due to the rapid development of chemoresistance.^6–8^

At the molecular level, PDAC is defined by recurrent genetic alterations—most notably KRAS activating mutations in over 90% of cases,^9^ and inactivation of key tumor suppressors such as TP53, CDKN2A, and SMAD4.^10,11^ These changes drive aberrant signaling through pathways such as MAPK, PI3K/AKT, and NF-κB, promoting proliferation and survival under cytotoxic stress.^12,13^ Concurrently, overexpression of ATP-binding cassette transporters actively exports chemotherapeutic agents, reducing intracellular drug accumulation and contributing substantially to resistance.^14^ Epigenetic reprogramming and dysregulated non-coding RNAs—such as miRNA-21, miRNA-145, and miRNA-155—further modulate gene expression to favor invasion, metastasis, and therapy escape.^15,16^

Extrinsic factors compound these intrinsic defenses. PDAC's hallmark desmoplastic stroma—composed of collagen, hyaluronan, cancer-associated fibroblasts, and infiltrating immune cells—creates a dense extracellular matrix that impedes drug penetration while secreting cytokines and growth factors that support tumor cell survival and resistance.^7,17,18^ Hypovascularity further limits agent delivery,^19^ and hypoxia within the tumor niche activates adaptive survival programs that blunt therapeutic efficacy.^20^

Addressing both intrinsic and extrinsic resistance mechanisms demands innovative approaches. Nanotechnology-based drug delivery systems have emerged as a promising strategy to surmount these barriers.^21,22^ By encapsulating chemotherapeutics within nanoscale carriers, these platforms improve drug stability, refine pharmacokinetics, and enable controlled, spatiotemporal release at the tumor site.^23–25^ Moreover, stimuli-responsive designs—triggered by acidic pH, elevated reducing environments, or specific enzymatic activity—ensure selective payload liberation within the tumor microenvironment.^26^ Surface functionalization with ligands or antibodies further enhances specificity, enabling precise delivery to both bulk tumor cells and resistant cancer stem cell populations.^27,28^

This review provides a focused analysis of the molecular and microenvironmental drivers of PDAC resistance and critically examines the latest nanomedicine strategies—ranging from smart, stimulus-responsive carriers to targeted, multifunctional platforms—designed to overcome these formidable challenges and improve clinical outcomes in pancreatic cancer.

## 2. Current landscape of drug resistance in pancreatic cancer

**Fig. 1:**
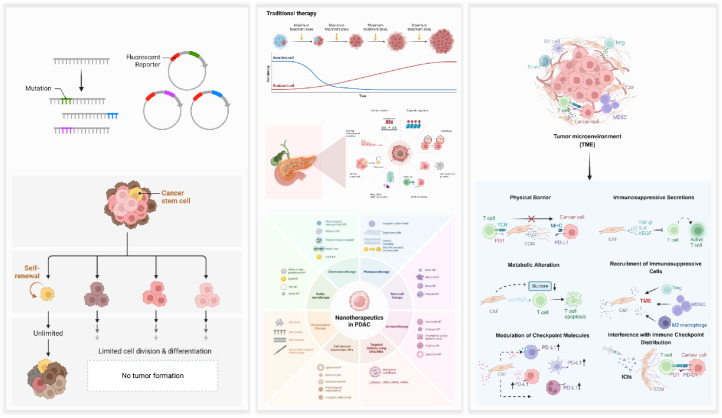
Overview of drug resistance mechanisms in pancreatic cancer created with https://www.BioRender.com.

Pancreatic cancer exhibits a remarkably complex array of resistance mechanisms that can be broadly classified into cell-autonomous (intrinsic) and microenvironment-driven (extrinsic) factors. As shown in Fig. 1, these mechanisms can be divided into intrinsic pathways, primarily involving genetic alterations, and extrinsic factors associated with the tumor microenvironment.

### 2.1 Intrinsic mechanisms

Genetic alterations are central to the inherent resistance observed in PDAC. Mutations in oncogenes such as KRAS, present in over 90% of cases,^9,29^ and tumor suppressor genes like TP53 and CDKN2A set the stage for a tumor phenotype that is highly resilient to chemotherapeutic insults.^30–32^ These mutations often lead to constitutive activation of survival pathways (*e.g.*, MAPK, PI3K/AKT, and NF-κB), thereby reducing apoptosis and promoting cell proliferation even in the presence of cytotoxic drugs.^33–37^ Moreover, PDAC cells often upregulate ATP-binding cassette (ABC) transporters, which actively pump chemotherapeutic agents out of the cells, further diminishing drug efficacy.^38,39^ Epigenetic changes^40,41^ and the modulation of non-coding RNAs (such as microRNAs-miRNA-21, miRNA-145, and miRNA-155)^42,43^ add another layer of complexity by altering the expression of genes involved in drug response and resistance. Cancer stem cells (CSCs) are also believed to contribute substantially to chemoresistance;^44,45^ these cells are characterized by high expression levels of drug efflux pumps, enhanced DNA repair capabilities, and a quiescent nature that makes them less susceptible to treatments that target rapidly dividing cells.^46–48^

### 2.2 Extrinsic mechanisms

The tumor microenvironment (TME) in PDAC is uniquely challenging. A hallmark of pancreatic tumors is an abundant desmoplastic stroma, comprising fibroblasts, immune cells, and a dense extracellular matrix (ECM) rich in collagen, fibronectin, and hyaluronan.^49^ This stroma creates significant physical barriers that impede the effective penetration of drugs.^35^ Additionally, the hypovascular and hypoxic nature of the TME further restricts drug delivery and fosters an immunosuppressive milieu.^50^ Immunosuppressive cells, including regulatory T cells (Tregs) and myeloid-derived suppressor cells (MDSCs), are recruited into the TME and secrete cytokines and growth factors that not only shield tumor cells from the immune system but also contribute to drug resistance by promoting survival signals.^51^

### 2.3 Rational design of responsive nanocarriers to overcome PDAC resistance

Pancreatic ductal adenocarcinoma (PDAC) employs intrinsic mechanisms, such as overexpression of ATP-binding cassette (ABC) transporters that efflux chemotherapeutics, and extrinsic barriers, including a desmoplastic stroma that hinders penetration and a hypoxic microenvironment that fosters survival, to resist treatment. Responsive nanocarriers have therefore been engineered with stimulus-triggered elements that directly counteract these defenses. Specifically, pH-sensitive micelles utilize acid-labile bonds that are stable at physiological pH (7.4) but cleave in acidic endo/lysosomal compartments (pH 6.5–5.5), achieving intracellular drug release that bypasses ABC transporter-mediated efflux.^52^ Redox-responsive systems incorporate disulfide linkages cleavable by elevated intracellular glutathione, ensuring payload stability during circulation and selective release within tumor cells.^53^ To overcome the stromal barrier, nanocarriers are surface-functionalized with peptide linkers that are degraded by matrix metalloproteinases (MMPs), enabling local degradation of collagen and hyaluronan to enhance penetration through the fibrotic matrix.^54^ By explicitly linking each design feature to the specific resistance mechanism it addresses, these strategies demonstrate that controlled release and active targeting are purposefully tailored to surmount PDAC's multifaceted drug resistance.

Collectively, the current landscape of drug resistance in pancreatic cancer paints a picture of a highly adaptive and multifactorial challenge. The convergence of genetic mutations, epigenetic reprogramming, enhanced efflux of drugs, and a complex, immunosuppressive microenvironment necessitates a shift towards more integrated and precision-based therapeutic strategies. Continued research into the molecular underpinnings of resistance is essential, as it will inform the design of next-generation therapies, such as nanomedicines and combination regimens, which may finally improve the prognosis for this formidable disease.

## 3. Innovative nanotechnology approaches targeting PDAC resistance

### 3.1 Overview of nanocarrier platforms and their impact on the therapeutic index

A diverse array of nanocarrier platforms, including liposomes, polymeric nanoparticles, dendrimers, micelles, and inorganic constructs, has been engineered to improve the pharmacokinetics and biodistribution of chemotherapeutics in PDAC.^55,56^ These systems leverage the enhanced permeability and retention (EPR) effect to preferentially accumulate in tumor tissue, protect labile drugs from premature degradation, and reduce off-target toxicity, as exemplified by liposomes encapsulating gemcitabine, which achieved a twofold increase in tumor drug levels alongside a 30 percent reduction in systemic myelosuppression compared with the free drug.^57^ While such enhancements to the therapeutic index are valuable, they do not directly overcome the three core resistance mechanisms of PDAC-ABC transporter efflux, cancer stem cell survival, and stromal barriers which drive disease relapse. The following subsections therefore focus on nanocarrier designs that explicitly target each of these challenges.

### 3.2 Inhibition of drug efflux transporters *via* stimuli-responsive release

Overexpression of ATP-binding cassette transporters such as P-glycoprotein and MRP1 actively expels chemotherapeutics from PDAC cells, undermining intracellular drug concentrations.^58,59^ To circumvent this defense, polymeric micelles bearing acid-labile bonds have been developed; these micelles remain stable at physiological pH (7.4) but undergo rapid bond cleavage within acidic endo/lysosomal compartments (pH 6.5–5.5), releasing over 80 percent of their payload within two hours at pH 5.5 while maintaining less than 10 percent leakage at pH 7.4, thereby delivering gemcitabine intracellularly faster than efflux pumps can remove it.^60^ In parallel, redox-responsive prodrugs exploit the millimolar intracellular glutathione gradient: doxorubicin linked *via* disulfide bonds exhibits a threefold higher intracellular retention under 10 mM glutathione compared to non-responsive controls, effectively overwhelming the MRP1-mediated efflux.^61^ By tying drug release to intracellular triggers, these platforms ensure cytotoxic concentrations are achieved inside PDAC cells despite elevated efflux activity.

### 3.3 Targeting cancer stem cells with functionalized nanocarriers

Cancer stem cells (CSCs) within PDAC, identified by surface markers such as CD44, CD24, and ESA, contribute to relapse through enhanced DNA repair, quiescence, and robust efflux capacity.^48^ To address this challenge, nanocarrier-based approaches have been developed to selectively target and eliminate CSCs. Functionalized nanoparticles conjugated with ligands or antibodies against CSC-specific markers have demonstrated improved uptake in stem-like cell populations and attenuated their ability to drive tumor progression.^62^ In addition to targeted delivery, co-loading of chemotherapeutics with agents that suppress stemness-related signaling pathways—such as small interfering RNAs (siRNAs) or pathway inhibitors—has been shown to enhance therapeutic efficacy and reduce the regenerative potential of CSCs.^63,64^ These strategies provide a promising avenue to overcome chemoresistance at its root by disrupting the CSC niche and limiting tumor repopulation.

### 3.4 Remodeling the tumor microenvironment to enhance penetration and reverse resistance

**Fig. 2:**
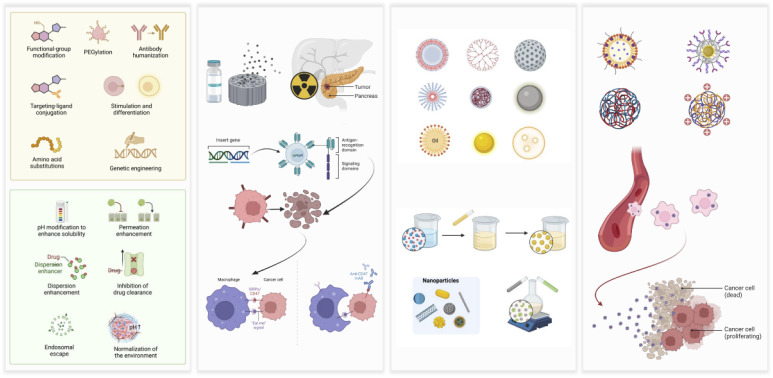
Innovative nanotechnology approaches in precision drug delivery active targeting: functionalization of nanoparticles (*e.g.*, liposomes and polymeric NPs) with ligands (*e.g.*, antibodies and peptides) for receptor-mediated uptake by tumor cells. Stimuli-responsive release: nanocarriers designed to release therapeutic payloads in response to tumor microenvironment triggers (*e.g.*, pH, enzymes, and redox gradients). Enhanced permeability and retention (EPR) effect: passive accumulation of nanoscale particles in tumor tissue due to leaky vasculature and impaired lymphatic drainage. Multifunctional systems: integration of imaging agents (*e.g.*, fluorescent dyes and contrast agents) for theranostic applications or combinatorial drug loading. Created with https://www.BioRender.com.

The tumor microenvironment (TME) in PDAC is characterized by a dense desmoplastic stroma, comprising extracellular matrix components such as collagen and hyaluronan, along with abundant cancer-associated fibroblasts.^65^ This structural complexity imposes significant physical and biochemical barriers to drug delivery, limiting the therapeutic impact of systemically administered agents. Nanotechnology-based strategies aimed at remodeling the TME have shown considerable potential in enhancing drug penetration and improving treatment responses.^66^ Responsive nanocarriers equipped with matrix metalloproteinase (MMP)-sensitive linkers can locally degrade stromal components, thereby facilitating deeper intratumoral distribution.^67^ Additionally, platforms that generate reactive oxygen or nitrogen species have been applied to modulate fibroblast activity, reduce interstitial fluid pressure, and normalize abnormal vasculature, collectively improving drug perfusion.^68^ By selectively altering the tumor stroma and microenvironmental conditions, these approaches enhance access to malignant cells and mitigate one of the most significant extrinsic mechanisms of drug resistance in PDAC (Fig. 2).

## 4. Mechanistic insights: nanomedicine interventions to counteract drug resistance

### 4.1 Bypassing cellular efflux and enhancing intracellular drug accumulation

**Fig. 3:**
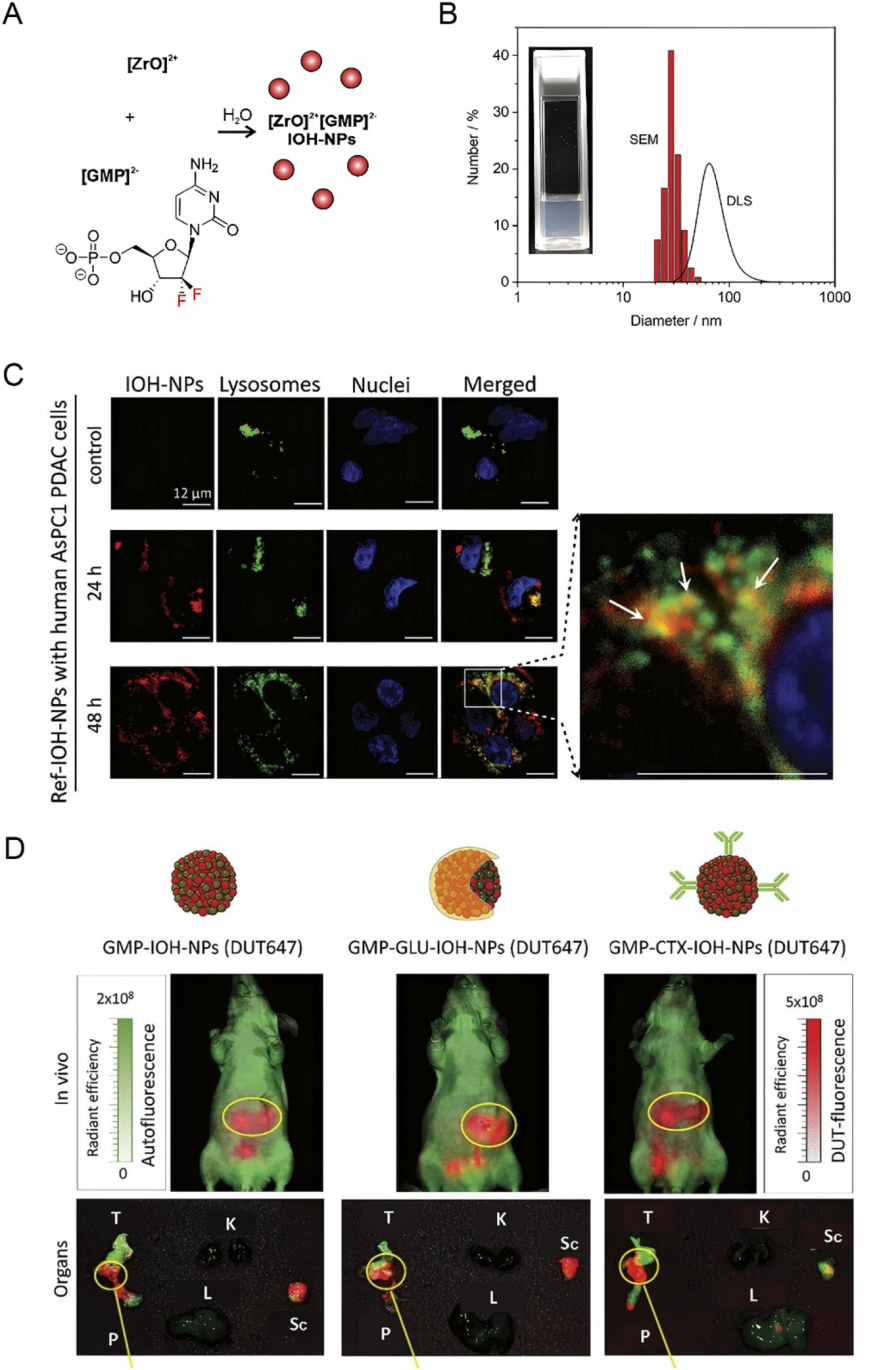
Synthesis, characterization, cellular uptake, and biodistribution of DUT647-Labeled Ref-IOH-NPs. (A) Scheme illustrating the aqueous synthesis; (B) particle size distribution according to DLS and SEM with a photograph of the aqueous suspension; (C) representative confocal fluorescence microscopy images showing the uptake of DUT647-labeled Ref-IOH-NPs after 24 and 48 h in AsPC1 cells co-stained with LysoTracker. The enlarged section shows GEM-free Ref-IOH-NPs colocalize in part with LysoTracker-labeled vesicles (yellow, marked with white arrows on the inset). Note that more LysoTracker-labeled vesicles are visible in comparison to the control without IOH-NPs (scale bars in a-c identical for all images); (D) spectral unmixing performed to distinguish between DUT647-derived fluorescence (red) and tissue autofluorescence (green). Reprinted with permission from ref. 71. Copyright @2023 Wiley.

Nanoparticles play a crucial role in drug delivery by bypassing cellular ABC transporter efflux mechanisms through receptor-mediated endocytosis, thereby increasing intracellular drug concentrations.^69,70^ Researchers have developed high drug-loading gemcitabine inorganic–organic hybrid nanoparticles (GMP-IOH-NPs) composed of [ZrO]^2+^ and gemcitabine monophosphate (GMP)^2−^, with GMP constituting 76% of the total nanoparticle mass^71^(Fig. 3). Self-assembled gemcitabine prodrug nanoparticles have demonstrated efficient cellular uptake and drug delivery capabilities, significantly increasing intracellular gemcitabine concentrations and overcoming chemoresistance.^72^ Additionally, gemcitabine-loaded chitosan nanoparticles have been shown to improve the drug's efficacy against pancreatic cancer cells, enhancing both apoptosis and ferroptosis responses.^73^ These nanoparticles enter tumor cells *via* receptor-mediated endocytosis, effectively circumventing ABC transporter efflux and enhancing intracellular drug levels. These advancements highlight the potential of nanoparticle-based delivery systems in enhancing the therapeutic efficacy of gemcitabine by overcoming drug resistance mechanisms and promoting cancer cell death through multiple pathways.

### 4.2 Overcoming the tumor microenvironment barrier

**Fig. 4:**
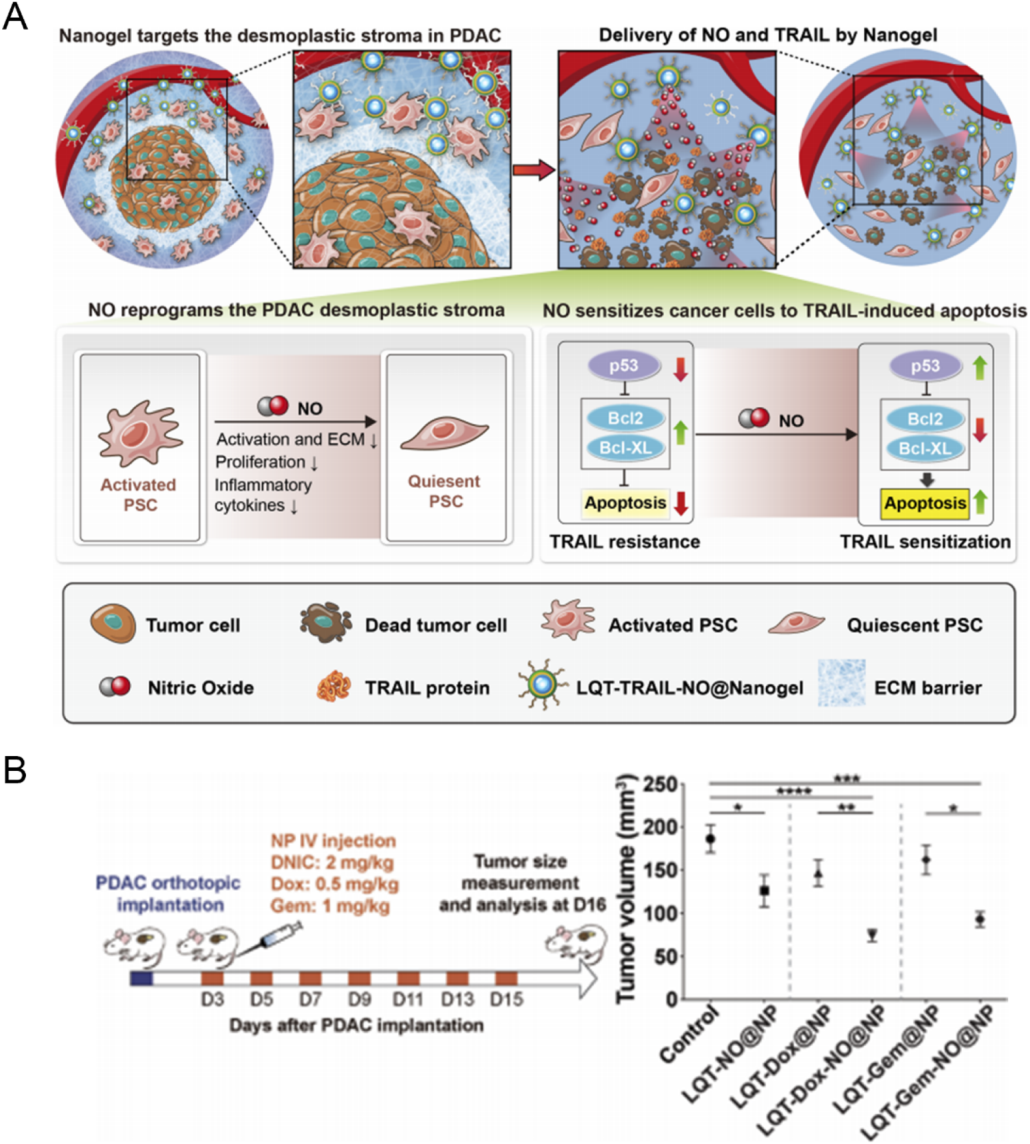
Tumor stroma-targeted TRAIL-NO@Nanogel remodels the fibrotic tumor microenvironment and suppresses PDAC progression in mice (A) schematic showing the mechanism by which tumour stroma-targeted TRAIL-NO@Nanogel suppresses PDAC progression in mice. NO released from tumour stroma-targeted TRAIL-NO@Nanogel remodels the fibrotic tumour microenvironment of desmoplastic PDAC; (B) three days after the implantation of AK4.4 PDAC cells and mice were treated with DNIC (2 mg kg^−1^) and/or Dox (0.5 mg kg^−1^) or Gem (1 mg kg^−1^) loaded in lipid-PLGA NPs modified with LQT28 on days 3, 5, 7, 9, 11, 13 and 15; tumours were then analysed on day 16. Volumes of orthotopic PDAC tumours 16 days post-implantation in treated and untreated (control) mice, DNIC: 2 mg kg; Dox: 0.5 mg kg; Gem: 1 mg kg^−1^. Reprinted with permission from ref. 77. Copyright @2021 Lippincott Williams and Wilkins Ltd.

The dense fibrotic stroma of pancreatic ductal adenocarcinoma (PDAC) significantly impedes drug penetration and induces hypoxic, nutrient-deprived conditions that activate tumor cell survival pathways, leading to drug resistance. Nanomedicine offers solutions by employing carriers capable of penetrating or modulating the tumor microenvironment (TME).^74,75^ Studies have demonstrated that nanoparticle-based photodynamic therapy (PDT) can effectively disrupt tumor–stroma interactions in pancreatic tumor models, sustainably inhibit extracellular matrix (ECM) secretion, and enhance therapeutic efficacy. For instance, photodynamic nanoparticles (UCNs@PPF), comprising upconversion nanoparticles (UCNs), protoporphyrin IX (PpIX), and polylysine (PLL)-modified folic acid (FA), generate reactive oxygen species (ROS) upon light irradiation. These ROS not only directly kill cancer cells and pancreatic stellate cells (PSCs) but also downregulate the TGF-β signaling pathway, reducing cancer cell proliferation and drug resistance.^76^ Additionally, researchers have developed tumor stroma-targeted nitric oxide (NO) nanogels for the co-delivery of NO and TRAIL. NO prevents tissue fibrosis, while TRAIL selectively induces apoptosis in cancer cells. These nanogels are functionalized with tumor stroma-targeting peptides identified through phage display technology, ensuring targeted delivery^77^(Fig. 4).

Targeting the TME barriers allows for a more comprehensive modulation of the TME, addressing multiple aspects of the stromal barrier simultaneously, and holds promise for further improving PDAC treatment outcomes.

### 4.3 Modulating intracellular signaling and gene expression

**Fig. 5:**
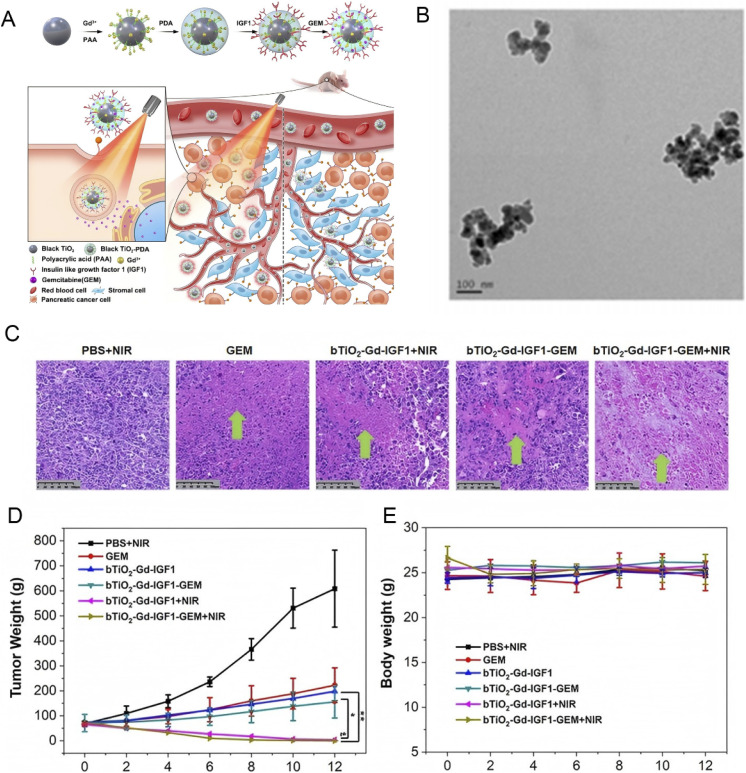
Synergistic photothermal-chemotherapy with GEM-loaded, IGF1-conjugated bTiO_2_ nanoprobes: TEM characterization, histological analysis, and *in vivo* efficacy in drug-resistant pancreatic cancer; (A) illustration of GEM loaded, IGF1 conjugated, black TiO_2_-based nanoprobes for 808 nm NIR triggered synergistic photothermal-chemotherapy in drug-resistant pancreatic cancer; (B) TEM images of bTiO_2_-Gd-IGF1; (C) tumor injury analysis *via* H&E staining following injection of mice with PBS, GEM, bTiO_2_-Gd-IGF1 and bTiO_2_-Gd-IGF1-GEM, followed by irradiation, in the presence or absence of for 5 min; (D) relative tumor volume and (E) mice body weights following the aforementioned treatments. Reprinted with permission from ref. 82. Copyright @2022 BioMed Central.

Cancer drug resistance primarily arises from genetic mutations and epigenetic modifications, which activate pro-survival pathways such as NF-κB, MAPK, and PI3K/AKT.^78–80^ Nanomedicine has shown tremendous potential in overcoming this challenge by delivering gene-silencing agents, such as siRNA, miRNA mimics, or antisense oligonucleotides, which precisely target these resistance mechanisms.^81^ By inhibiting the expression of resistance-related genes, nanocarriers can resensitize cancer cells to conventional therapies. For example, Xu *et al.* provided a successful case by developing black titanium dioxide (bTiO_2_) nanoprobes. Under the synergistic action of photothermal therapy and chemotherapy, these nanoprobes not only disrupt the tumor stromal barrier but also reverse drug resistance. Their *in vitro* and *in vivo* experiments demonstrated that the nanoprobes significantly enhanced the efficacy of gemcitabine, nearly eliminating resistant pancreatic tumors^82^(Fig. 5).

Together, these approaches not only disrupt the tumor microenvironment physically (*e.g.*, through photothermal therapy) to improve drug penetration but also act at the gene regulation level by targeting specific signaling pathways and gene expressions to reverse resistance. Such findings provide critical scientific evidence and practical guidance for developing more effective treatment strategies for pancreatic cancer.

### 4.4 Synergistic combination approaches

**Fig. 6:**
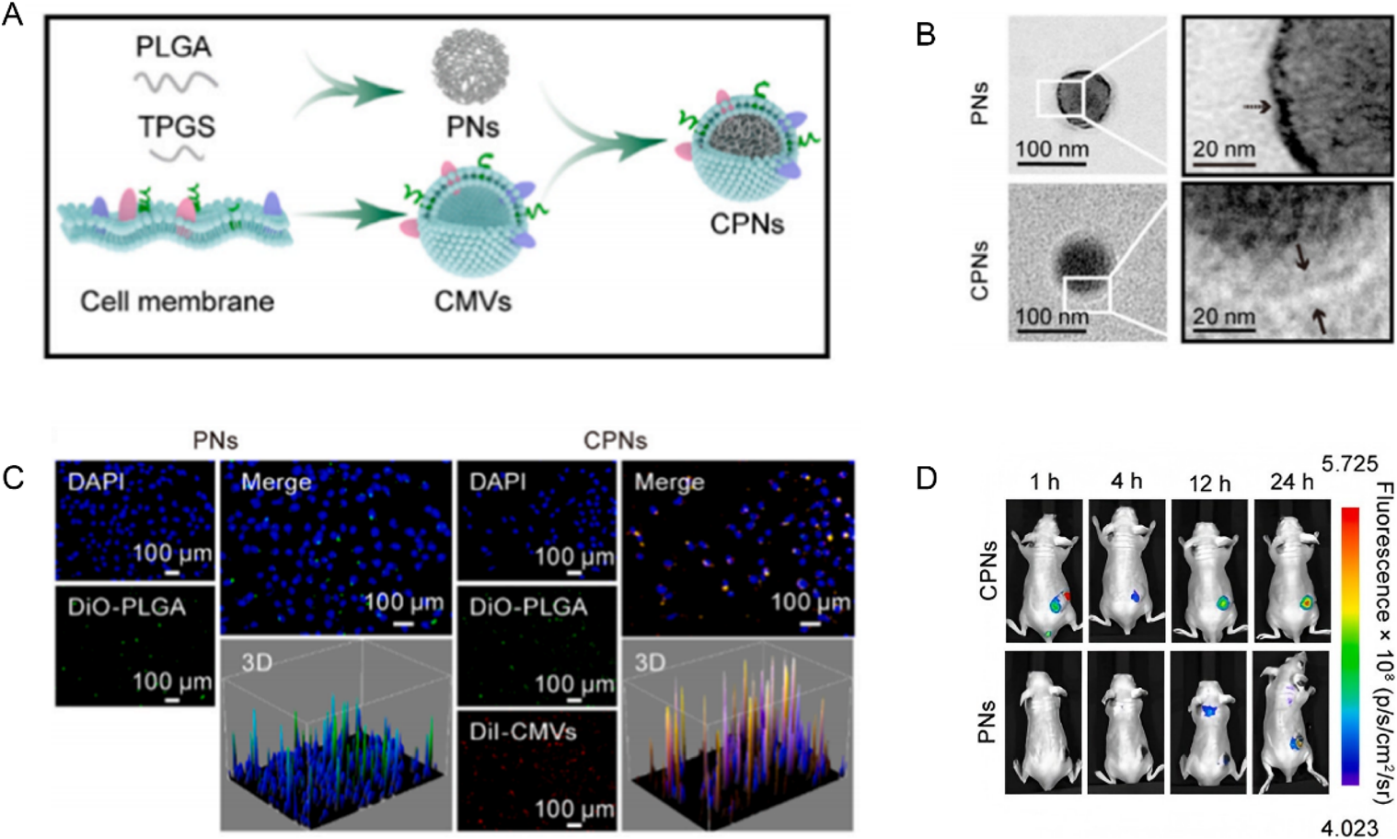
Development and characterization of a cancer cell membrane-camouflaged BA delivery system with enhanced tumor targeting (A) the cell membrane-camouflaged delivery system for BA was developed using PLGA, TPGS, and cancer cell membrane; (B) TEM was used to characterize the morphology of PNs, CMVs (positive control), and CPNs; (C) fluorescence microscopy showed that compared to PNs (green dots), more CPNs (yellow dots) were absorbed by tumor cells; (D) *in vivo* fluorescence images proved that CPNs are superior to PNs in the delivery of BA to the tumor sites. Reprinted with permission from ref. 85. Copyright @2023 Elsevier.

Integrating nanomedicine with conventional therapies offers a multifaceted approach to overcoming drug resistance. For example, co-delivery systems can simultaneously transport chemotherapeutic agents and survival pathway inhibitors to achieve synergistic effects.^83,84^ These integrated platforms not only enhance the efficacy of the primary drug but also suppress the compensatory mechanisms cancer cells use to evade treatment. Multifunctional nanoparticles that combine therapeutic and diagnostic capabilities (theranostics) enable clinicians to monitor treatment responses in real time and adjust dosing or combination regimens accordingly^85^(Fig. 6).

**Fig. 7:**
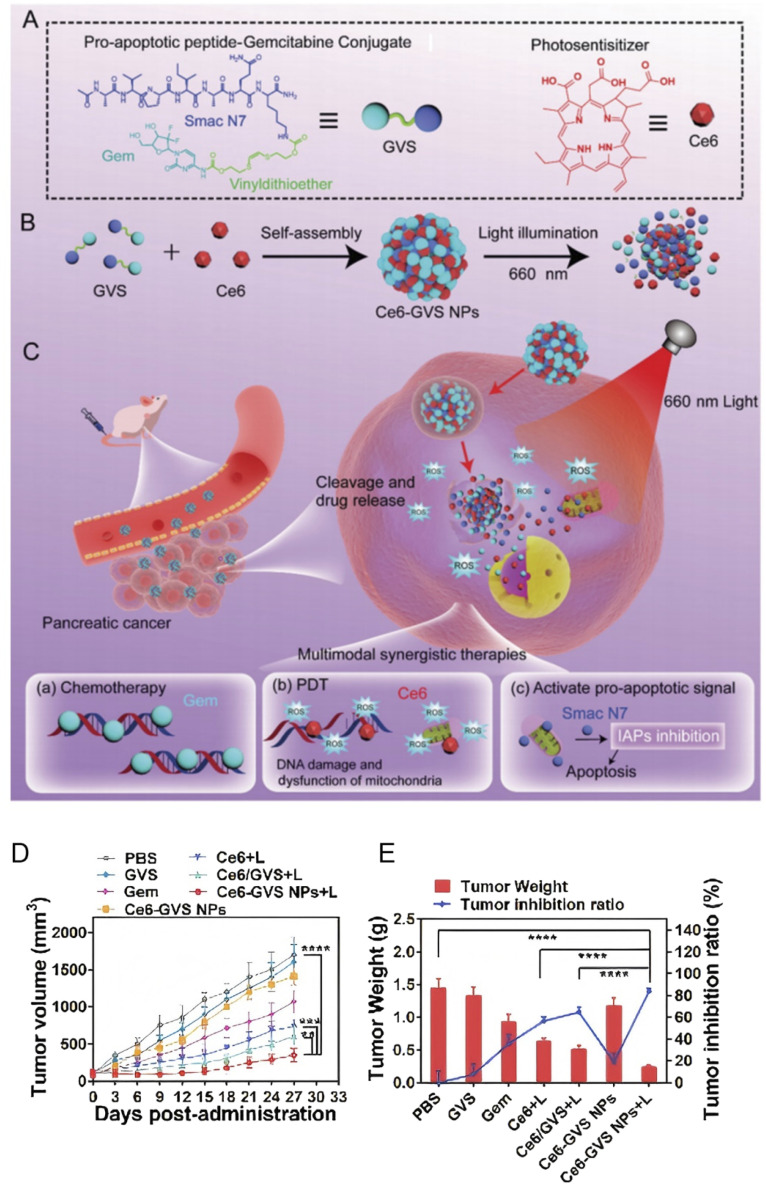
Development and evaluation of Ce6-GVS nanoparticles: chemical structures, self-assembly process, therapeutic mechanisms, and antitumor efficacy (A) chemical structures of Ce6 and GVS; (B) the preparation of Ce6-GVS NPs by the self-assembly of Ce6 and GVS; (C) therapeutic pathways and mechanisms of the nanomedicine; (D) tumor burden after various treatments; (E) excised tumor weights and the corresponding tumor inhibition ratios after 27 days of treatment in different groups. Reprinted with permission from ref. 86. Copyright @2022 Royal society of chemistry.

Among numerous studies, one nanomedicine platform that combines photodynamic therapy (PDT) and chemotherapy has demonstrated significant synergistic antitumor effects. This platform employs the self-assembly of Chlorin e6 (Ce6) with a prodrug containing the pro-apoptotic peptide Smac N7 and gemcitabine (Gem). Under light irradiation, the system triggers rapid release of Gem, Ce6, and Smac N7, along with the generation of reactive oxygen species (ROS). The ROS disrupt the tumor cell's antioxidant defenses, while Smac N7 inhibits inhibitors of apoptosis proteins (IAPs), thereby enhancing the cytotoxicity of Gem. This synergistic interaction not only boosts the overall therapeutic efficacy but also markedly reduces tumor cell resistance^86^(Fig. 7).

The strength of multifunctional nanoparticles lies in their dual capacity for therapy and diagnosis. By integrating Ce6, Gem, and Smac N7, these nanomedicines can monitor treatment responses in real time and adjust drug release based on changes in the tumor microenvironment. This intelligent drug delivery system addresses the challenges of uneven drug distribution and resistance seen in traditional therapies, providing a promising new strategy for treating pancreatic cancer.

### 4.5 Immune modulation and microenvironment reprogramming

**Fig. 8:**
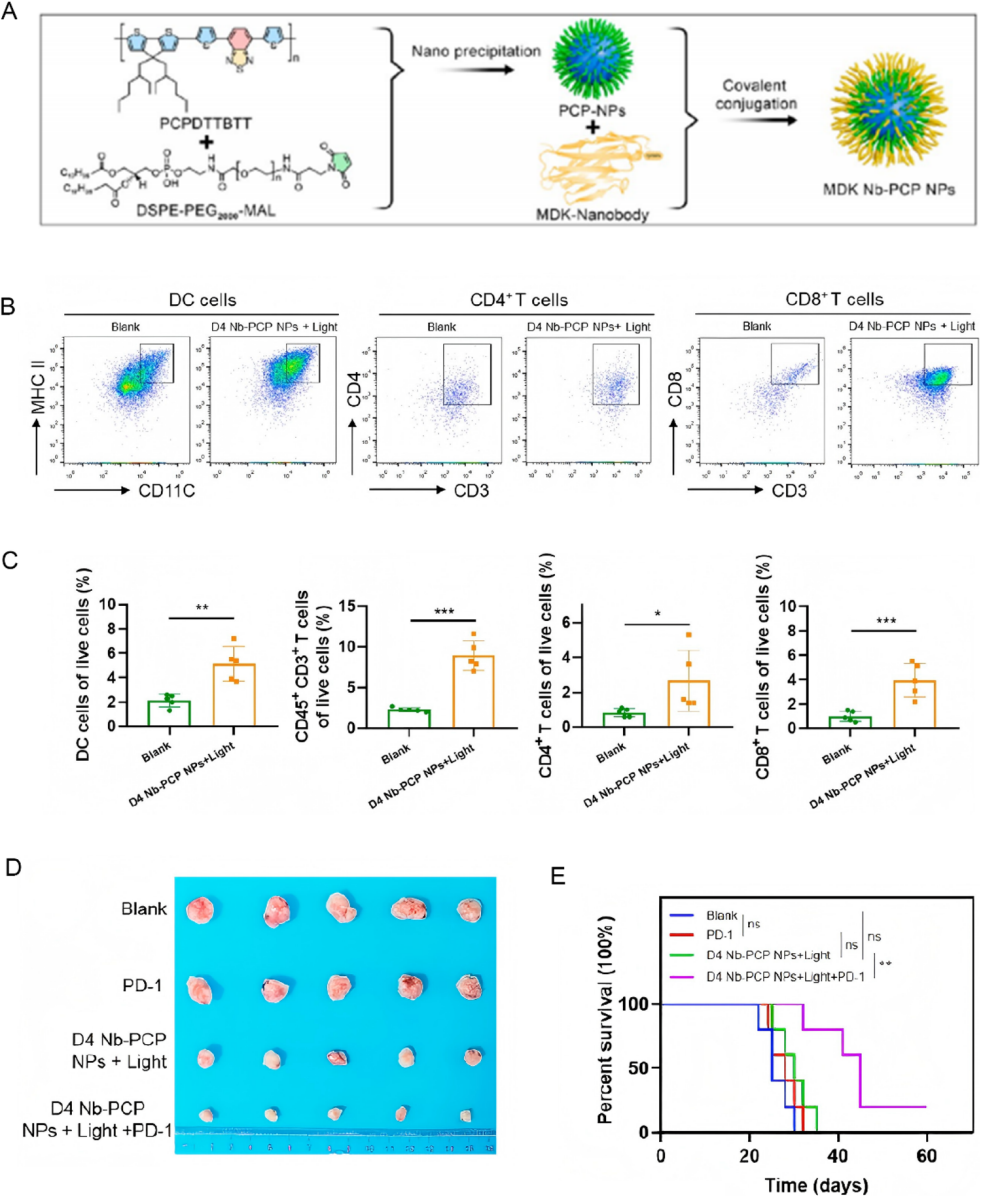
Synthesis and immunotherapeutic efficacy of MDK Nb-PCP nanoparticles: impact on dendritic cell maturation, T cell infiltration, tumor regression, and survival outcomes; (A) flowchart of the synthesis of MDK Nb-PCP NPs; (B) the analysis of DC cells and the examination of CD4+ T cells and CD8+ T cells in the two groups; (C) corresponding quantitative analysis of mature DC cells, CD45+ CD3+ T cells, CD4+ T cells, and CD8+ T cells in the two groups; (D) tumor appearance after different treatments; (E) the survival analysis of mice in different groups. Reprinted with permission from ref. 90. Copyright @2024 American Chemical Society.

**Fig. 9:**
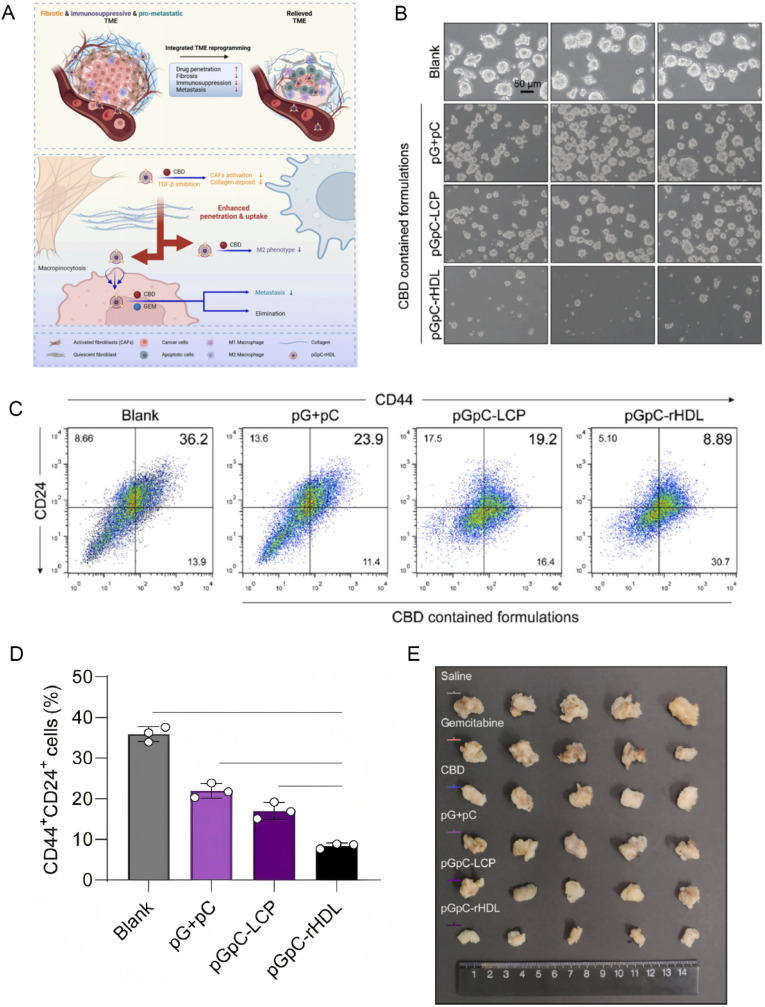
pGpC-rHDL remodels the PDAC tumor microenvironment to inhibit tumor stemness and progression; (A) schematic illustration of the biomimetic rHDL (pGpC-rHDL) attenuating the fibrotic, immunosuppressive and pro-metastatic TME and suppressing the PDAC progression; (B) representative morphology of KPC1199 tumor spheres after treatment with pG + pC, pGpC-LCP or pGpC-rHDL (equiv. CBD of 5 μM, gemcitabine of 0.5 μM) for 24 h. Scale bar, 50 μm; (C) the flow cytometry of CD44+/CD24+ cells collected from tumor spheres; (D) quantitative analysis of the CD44+/CD24+ cells; (E) images of the excised orthotopic tumors in each group on the day after the last administration. Reprinted with permission from ref. 97. Copyright @2025 Elsevier.

Beyond direct cytotoxic effects, nanomedicine can alter the immunosuppressive landscape of the tumor microenvironment.^87^ Nanoparticles engineered to deliver immune adjuvants or checkpoint inhibitors locally can reprogram the tumor milieu to support immune cell infiltration and activation.^88^ By disrupting the protective niche that cancer cells construct, through modulation of stromal cells and cytokine secretion, these interventions can render previously “cold” tumors more immunogenic and responsive to immunotherapy, thereby addressing another layer of drug resistance.^89^ Research has shown that precise photodynamic therapy (PDT) using Midkine-targeted, nanobody-engineered nanoparticles can effectively remodel the tumor microenvironment (TME) of pancreatic ductal adenocarcinoma (PDAC) and activate anti-tumor immune responses. Specifically, under light irradiation, D4 Nb-PCP nanoparticles generate abundant reactive oxygen species (ROS), which induce immunogenic cell death (ICD) in tumor cells, evidenced by the translocation of CRT and ERp57 to the cell membrane and increased HMGB1 secretion. These changes stimulate the immune system to recognize and attack the tumor. Additionally, after PDT, there is an increase in mature dendritic cells and enhanced infiltration of CD4+ and CD8+ T cells within the PDAC TME, indicating a shift from an immunosuppressive to an immunoactive state. Elevated levels of pro-inflammatory cytokines in the tumor tissue further confirm the remodeling of the immune microenvironment. Ultimately, when combined with PD-1 antibody therapy, this precise PDT significantly enhances anti-tumor efficacy and prolongs survival in mice, offering a promising new strategy for PDAC immunotherapy^90^ (Fig. 8). A major contributor to therapeutic failure is the presence of cancer stem cells, which possess intrinsic resistance mechanisms including enhanced drug efflux, robust DNA repair, and quiescence.^91,92^ Nanomedicine interventions have been tailored to specifically target CSC markers (such as CD44) to eliminate this resistant cell subpopulation.^93,94^ Functionalized nanocarriers, such as the aforementioned anti-CD44-conjugated nanocapsules, have demonstrated significant efficacy in selectively delivering cytotoxic drugs to CSCs, thereby reducing the likelihood of tumor relapse and metastasis.^95,96^ A study developed a PDAC-specific, nutrient-mimetic recombinant high-density lipoprotein (rHDL), termed pGpC-rHDL, which effectively remodels the tumor microenvironment (TME) of pancreatic ductal adenocarcinoma (PDAC). It achieves this by inhibiting the activation of cancer-associated fibroblasts (CAFs), reducing collagen deposition, and suppressing both M2 macrophage polarization and the expression of the immunosuppressive cytokine IL-6. Notably, pGpC-rHDL significantly impedes pancreatic tumor spheroid formation and lowers the proportion of CD44^+^/CD24^+^ cell subpopulations, indicating its potential to effectively downregulate tumor cell stemness^97^(Fig. 9).

Together, these mechanistic insights illustrate that nanomedicine does more than simply transport drugs; it actively intervenes in the molecular and cellular pathways that underlie drug resistance. By enhancing intracellular drug delivery, modulating the tumor microenvironment, reprogramming genetic and epigenetic regulators, and targeting resilient cancer stem cells, innovative nanotechnology strategies hold the promise of transforming the treatment landscape for pancreatic cancer and other recalcitrant malignancies.

## 5. Revolutionizing target discovery: integrative omics and AI-driven nanomedicine for personalized pancreatic cancer therapy

### 5.1 Spatial and single-cell omics breakthroughs

Cutting-edge single-cell and spatial omics techniques now enable the dissection of tumor heterogeneity at unprecedented resolution.^98^ By mapping gene expression and protein distributions within individual cells and their spatial contexts, researchers can identify rare but critical subpopulations, such as cancer stem cells and resistant clones. This level of granularity reveals unique biomarkers and vulnerabilities that can be exploited through nanotherapeutics, ensuring that treatment is tailored not only to the dominant tumor type but also to its most elusive, drug-resistant niches.^99^

### 5.2 Systems biology and multi-layer omics integration

Integrative analyses that combine genomics, transcriptomics, proteomics, metabolomics, and even epitranscriptomics create comprehensive molecular portraits of pancreatic cancer.^100^ These systems biology approaches uncover complex networks and feedback loops that drive tumor progression and drug resistance. By synthesizing multi-dimensional data, researchers can pinpoint key regulatory nodes and signaling pathways, paving the way for the design of nanocarriers that specifically target these critical hubs, thereby disrupting the tumor's survival strategies.

### 5.3 AI-driven data mining and predictive modeling

The advent of machine learning and artificial intelligence has revolutionized omics data analysis. Advanced algorithms can sift through massive datasets to identify novel biomarkers and predict the functional consequences of specific mutations.^101,102^ These insights not only inform the selection of therapeutic targets but also guide the customization of nanotherapeutics. AI-driven predictive models facilitate the design of smart, adaptive nanoparticles that adjust drug release profiles in real time based on the tumor's evolving molecular landscape.^103^

### 5.4 Dynamic, real-time monitoring with theranostic nanoplatforms

Integration of omics data with nanotechnology has opened up the field of theranostics where treatment and diagnostics converge. Nanocarriers equipped with imaging agents and biosensors enable real-time monitoring of treatment efficacy and the tumor's molecular response. This dynamic feedback allows clinicians to modify treatment regimens on the fly, ensuring that nanotherapeutic interventions remain effective even as the tumor evolves and develops new resistance mechanisms.^104^

### 5.5 Personalized nano-targeting based on omics signatures

The ultimate goal of integrating multi-omics data is to enable truly personalized therapy. By correlating specific omics signatures with drug response profiles, clinicians can stratify patients and design individualized treatment protocols. Nanocarriers can be functionalized with ligands that precisely target biomarkers identified through omics analyses, ensuring that each patient receives a custom-tailored therapy optimized to overcome their tumor's unique resistance patterns.

Collectively, these innovative approaches underscore the potential of integrating multi-omics data, advanced AI analytics, and smart nanotherapeutics to revolutionize target discovery and treatment personalization in pancreatic cancer. This convergence not only enhances our understanding of tumor biology but also sets the stage for the next generation of precision medicine strategies aimed at overcoming drug resistance. As summarized in Table 1, integrating multi-omics data, advanced AI analytics, and smart nanotherapeutics offers a revolutionary approach to personalized pancreatic cancer therapy.

**Table 1:** Summary of integrative omics and AI-driven nanomedicine approaches for personalized pancreatic cancer therapy

Technological approach	Key insights	Nanomedicine application
Single-cell and spatial omics techniques	Detailed mapping of tumor heterogeneity; identification of rare subpopulations (*e.g.*, cancer stem cells and resistant clones)	Tailoring nanotherapeutics to target elusive, drug-resistant niches and specific cellular subpopulations
Integrative analysis of genomics, transcriptomics, proteomics, metabolomics, and epitranscriptomics	Comprehensive molecular portraits; uncovering complex networks, regulatory nodes, and feedback loops	Designing nanocarriers that precisely target key signaling pathways and regulatory hubs driving tumor progression and resistance
Machine learning and artificial intelligence algorithms	Identification of novel biomarkers; prediction of functional consequences of mutations	Guiding the customization of smart, adaptive nanoparticles that adjust drug release profiles based on the tumor's evolving molecular landscape
Integration of omics data with imaging agents and biosensors embedded in nanocarriers	Real-time monitoring of treatment efficacy and tumor molecular responses	Enabling theranostic platforms that provide dynamic feedback to adjust treatment regimens in real time, ensuring sustained therapeutic effectiveness
Correlating multi-omics signatures with drug response profiles	Patient stratification; identification of individualized biomarkers	Functionalizing nanocarriers with specific ligands to achieve highly personalized therapy tailored to each patient's unique molecular profile

## 6. Discussion and future perspectives

Nanotechnology is redefining pancreatic cancer treatment by addressing the notorious drug resistance seen in traditional therapies. Recent advancements in nanomedicine have led to the development of various nanocarriers, such as liposomes, polymeric nanoparticles, dendrimers, and inorganic nanomaterials, which improve drug stability, control release, and enhance pharmacokinetics. However, given the heterogeneous nature of pancreatic ductal adenocarcinoma (PDAC), future research must focus on creating multifunctional, adaptive nanoparticles capable of responding to local stimuli like pH changes, redox conditions, or enzymatic activity. These smart systems, equipped with on-demand drug release triggered by biosensor feedback, promise to deliver high concentrations of therapeutic agents precisely when needed.

Overcoming the formidable tumor microenvironment is another critical challenge. The dense stroma in PDAC not only limits drug penetration but also fosters an immunosuppressive, hypoxic milieu. Nanoparticles that remodel this environment, by normalizing vasculature or degrading extracellular matrix components, could work synergistically with traditional chemotherapies and immunotherapies. Additionally, addressing cellular resistance mechanisms through the targeted delivery of gene-silencing molecules or combination therapies that interfere with key survival pathways is essential.

The integration of diagnostic and therapeutic functions in theranostic nanoparticles further enables real-time monitoring of treatment responses, paving the way for personalized therapies. Despite these promising developments, issues related to the scalable synthesis, long-term safety, and regulatory approval of these nanoplatforms remain. Continued interdisciplinary collaboration and standardized preclinical models will be vital for translating these innovative strategies from the laboratory to clinical practice.

## 7. Conclusions

Nanotechnology-based approaches offer a transformative avenue for overcoming the notorious drug resistance observed in pancreatic cancer. By harnessing the unique properties of nanocarriers, including enhanced stability, controlled release, and active targeting, researchers have developed innovative strategies that address both intrinsic and extrinsic mechanisms of resistance. These advanced platforms not only improve the intracellular delivery of therapeutic agents by bypassing efflux pumps and protecting drugs from premature degradation but also modulate the tumor microenvironment to facilitate drug penetration and re-sensitize resistant cancer cells. Although significant progress has been made, translating these promising strategies into clinical practice remains a formidable challenge. Continued interdisciplinary research, coupled with the integration of real-time monitoring and personalized treatment protocols, is essential to fully exploit the potential of nanomedicine. As we refine these technologies and address the remaining hurdles, nanotechnology is poised to redefine precision medicine in pancreatic cancer, offering new hope for improved outcomes and a better quality of life for patients battling this aggressive disease.

## Author contributions

Linjia Peng and Yanfeng Liang conceptualized the article and wrote the draft of the manuscript. Qiuli Zhang, Xiaonan Guo, Zixuan Gao, Xinxin Kong, and Haiting Zhang contributed to the writing of specific sections and conducted a thorough review and revision of the manuscript. Binyu Zhu and Daxiang Cui reviewed and revised the manuscript. All authors contributed to the article and approved the submitted version. Qiuli Zhang, Xiaonan Guo, Zixuan Gao, Xinxin Kong, and Haiting Zhang developed the methodology, conducted formal analysis, and contributed to data curation and visualization. Binyu Zhu prepared figures and assisted with manuscript editing. Daxiang Cui supervised the project, provided critical revisions, and secured funding. All authors reviewed and approved the final manuscript.

## Conflicts of interest

All authors declare no conflict of interest.

## Data Availability

The data supporting the findings of this review are derived from publicly available sources cited throughout the manuscript. All referenced studies and datasets can be accessed through their respective publications or repositories.
